# Phase Transitions in the “Spinel-Layered” Li_1+x_Ni_0.5_Mn_1.5_O_4_ (x = 0, 0.5, 1) Cathodes upon (De)lithiation Studied with Operando Synchrotron X-ray Powder Diffraction

**DOI:** 10.3390/nano11061368

**Published:** 2021-05-21

**Authors:** Oleg A. Drozhzhin, Anastasia M. Alekseeva, Vitalii A. Shevchenko, Dmitry Chernyshov, Artem M. Abakumov, Evgeny V. Antipov

**Affiliations:** 1 Department of Chemistry, Lomonosov Moscow State University, 119991 Moscow, Russia; alekseevaAM@gmail.com (A.M.A.); shev110195@rambler.ru (V.A.S.); evgeny.antipov@gmail.com (E.V.A.); 2 Skoltech Center for Energy Science and Technology, Skolkovo Institute of Science and Technology, Nobel str. 3, 143026 Moscow, Russia; a.abakumov@skoltech.ru; 3 Swiss–Norwegian Beamlines, European Synchrotron, 71 Rue des Martyrs, 38043 Grenoble, France; dmitry.chernyshov@esrf.fr; 4 Institute of Physics, Nanotechnology and Telecommunications, Peter the Great St. Petersburg Polytechnic University, 29 Polytekhnicheskaya St., 195251 Saint-Petersburg, Russia

**Keywords:** Li-ion, cathode material, oxides, spinel, LiNi_0.5_Mn_1.5_O_4_, spinel-layered composite, high-voltage

## Abstract

“Spinel-layered” Li_1+*x*_Ni_0.5_Mn_1.5_O_4_ (*x* = 0, 0.5, 1) materials are considered as a cobalt-free alternative to currently used positive electrode (cathode) materials for Li-ion batteries. In this work, their electrochemical properties and corresponding phase transitions were studied by means of synchrotron X-ray powder diffraction (SXPD) in operando regime. Within the potential limit of 2.2–4.9 V vs. Li/Li^+^ LiNi_0.5_Mn_1.5_O_4_ with cubic spinel type structure demonstrates the capacity of 230 mAh·g^−1^ associated with three first-order phase transitions with significant total volume change of 8.1%. The Li_2_Ni_0.5_Mn_1.5_O_4_ material exhibits similar capacity value and subsequence of the phase transitions of the spinel phase, although the fraction of the spinel-type phase in this material does not exceed 30 wt.%. The main component of Li_2_Ni_0.5_Mn_1.5_O_4_ is Li-rich layered oxide Li(Li_0.28_Mn_0.64_Ni_0.08_)O_2_, which provides nearly half of the capacity with very small unit cell volume change of 0.7%. Lower mechanical stress associated with Li (de)intercalation provides better cycling stability of the spinel-layered complex materials and makes them more perspective for practical applications compared to the single-phase LiNi_0.5_Mn_1.5_O_4_ high-voltage cathode material.

## 1. Introduction

Since the discovery of extraordinary electrochemical capacity for so-called “Li-rich” oxides as positive electrodes for Li-ion batteries, materials exploiting anionic redox activity have been the subject of intense studies [1,2,3,4,5]. Mn- and Li-rich NMCs with a typical Li[Li,Mn,Ni,Co]O_2_ composition were firstly regarded as two-phase composites consisting of the layered rhombohedral (s.g. *R*$\bar{3}$*m*) phase Li(Mn,Ni,Co)O_2_ and the layered monoclinic phase Li_2_MnO_3_ (s.g. *C*2/*m*) as nano-sized domains, while later Li-rich NMCs were recognized as single-phase materials with local cation ordering. It is worth noting that pure Li_2_MnO_3_ was considered to be electrochemically inactive for a long time, before demonstration of reversible Li^+^ extraction/insertion after “activation” at ~4.5–4.7 V vs. Li/Li^+^ in highly dispersed powder [6]. Unlike the LiMO_2_ layered oxides (M—transition metal), Li_2_MnO_3_ (also represented as Li[Li_1/3_Mn_2/3_]O_2_) exhibits oxygen anion redox activity that has been confirmed by a number of experimental and computational studies [7,8,9]. Single-phase Li_2_MnO_3_ suffers from poor cycling stability, low Coulombic efficiency, voltage fading, etc. On the other side, monoclinic Li-rich NMCs exhibit much better capacity retention during cycling demonstrating the capacity up to 300 mAh·g^−1^. However, in spite of large reversible capacity, Li-rich NMCs still have not found an application because of well-known drawbacks such as irreversible d-cation migration, leading to spinel phase formation and voltage fading [5,10,11,12,13,14].

Another promising direction for the development of oxide materials for Li-ion batteries are a high-voltage spinel LiNi_0.5_Mn_1.5_O_4_ and “spinel-layered” materials demonstrating attractive electrochemical performance with ~700–800 Wh·kg^−1^ of the cathode energy density [15,16,17,18]. Their nominal composition can be written as Li_1+*x*_Ni*_y_*Mn_2–*y*_O_4_ and *x* value typically determines the spinel to layered phase ratio. When *x* = 0 and *y* = 0.5, the well-known high-voltage spinel LiNi_0.5_Mn_1.5_O_4_ is formed [19]. Increasing *x* by using the excess amount of lithium source for the synthesis leads to monoclinic layered oxide as a second phase. Additionally, an impurity Li*_z_*Ni_2−*z*_O_2_ phase typically associated with a nickel-rich rock-salt derivative is often observed [15,16,17,18]. It should be noted that chemical or electrochemical lithiation of LiNi_0.5_Mn_1.5_O_4_ results in a tetragonal Li_2_Ni_0.5_Mn_1.5_O_4_ phase (s.g. *I*4_1_/*amd*), so the Li_1+*x*_Ni_0.5_Mn_1.5_O_2_ oxides demonstrate path-dependent phase composition [20,21,22]. Authors who studied electrochemical behavior of the “spinel-layered” composites pointed out high reversible capacity (>200 mAh/g) and excellent cycling stability of these materials, exceeding performance of single-phase LiNi_0.5_Mn_1.5_O_4_. Ex situ X-ray diffraction and microscopy studies revealed the presence of both monoclinic (layered) and cubic (spinel) phases after long-term cycling; however, there is lack of data concerning changes occurring directly during (De)lithiation of “spinel-layered” composite materials [13,14,15,16,17,18].

The study of phase and structural transformations taking place in electrode materials during electrochemical charge–discharge is vital for understanding the processes underlying battery functioning, which, in turn, is necessary for the development of new materials and technologies. Phase transitions occurring during lithiation and de-lithiation of LiNi_0.5_Mn_1.5_O_4_ have already been described [23,24,25,26]. There are two two-phase transitions at the high-voltage part (ca. ~4.7 V vs. Li/Li^+^), conserving spinel-type structure and cubic-to-tetragonal transition at the low-voltage plateau (ca. 2.8 V vs. Li/Li^+^). Structural changes in Li-rich NMCs were also studied in a number of papers, including oxygen evolution during first charge, activation of Li in Li-TM (transition metal) layer, migration of TM into Li positions and spinel formation during cycling [5,9,10,11,12,13,14]. On the other hand, the processes occurring in “spinel-layered” Li_1+*x*_Ni_0.5_Mn_1.5_O_4_ composites during electrochemical cycling are still unclear and have not been studied so far. The main goal of our work was to fill this gap.

Thus, we report here synthesis, electrochemical characterization and detailed study of the phase transitions in Li_1+*x*_Ni_0.5_Mn_1.5_O_4_ (*x* = 0, 0.5, 1) cathode materials by means of operando synchrotron X-ray powder diffraction (SXPD).

## 2. Materials and Methods

The samples of the Li_1+*x*_Ni_0.5_Mn_1.5_O_4_ (*x* = 0, 0.5, 1.0) nominal compositions were prepared using hydrothermal synthesis as described previously [27,28]. Briefly, NiSO_4_·6H_2_O and MnSO_4_·H_2_O were dissolved in water in molar ratio of 1:3. Sodium carbonate solution was added and the obtained suspension was heated to 140 °C and conditioned at this temperature for 12 h. The precipitate was washed, dried and mixed with LiOH taken with 5% excess. The precursor was annealed at 350 °C for 2 h and then at 800 °C for 10 h with intermediate regrinding.

The phase composition of the samples was characterized using powder X-ray diffraction (PXRD, Huber Guinier camera G670, CuK_α1_ radiation, λ = 1.5406 Å). The particle size and morphology were studied using a JEOL JSM-6490LV (30 kV, W-cathode) scanning electron microscope (SEM).

Samples for the transmission electron microscopy (TEM) study were prepared by crushing the materials in an agate mortar under ethanol and depositing a few drops of suspension onto copper grids covered by a holey carbon layer. High angle annular darkfield scanning transmission electron microscopy (HAADF-STEM) images and energy-dispersive X-ray (EDX) spectra were collected with a probe aberration-corrected FEI Titan G3 microscope operated at 200 kV and equipped with a Super-X EDX system.

Electrode materials for electrochemical testing were prepared by mixing 80 mass.% of active compound, 10 mass.% of carbon black and 10 mass.% of polyvinylidene fluoride (PVDF) binder in N-methylpyrrolidone followed by spreading the obtained mixture on aluminum foil by doctor blade technique. Dried electrodes were calendered, punched to round discs and dried at 110 °C for 3 h under dynamic vacuum. Two-electrode cells were assembled in an Ar-filled glove-box (MBraun). Lithium metal was used as the counter electrode and 1 M solution of LiBF_4_ in sulfolane was used as the electrolyte. All galvanostatic experiments were carried out using Elins P-20X8 potentiostat-galvanostat (ES8 software) within 2.2–4.9 V vs. Li/Li^+^.

Synchrotron X-ray powder diffraction (SXPD) in operando regime was performed at the Swiss Norwegian Beamlines (SNBL), BM01 and BM31, at the European Synchrotron Radiation Facility (ESRF, Grenoble, France). The original electrochemical cell with single-crystal sapphire X-ray windows was used [29]. Experiments were conducted in a low intensity beam mode (~40 mA, 4 × 10 filling mode). The PILATUS@SNBL diffractometer was used for SXPD studies (λ = 0.7225 Å). The 2D diffraction data from a Pilatus 2 M detector were processed using the SNBL Toolbox and BUBBLE software [30]. The time of data acquisition was 10 s per pattern. Rietveld refinement using RIETAN-FP software was applied to analyze SXPD patterns [31]. In some cases, Jana 2006 software was applied [32].

## 3. Results

**Figure 1:**
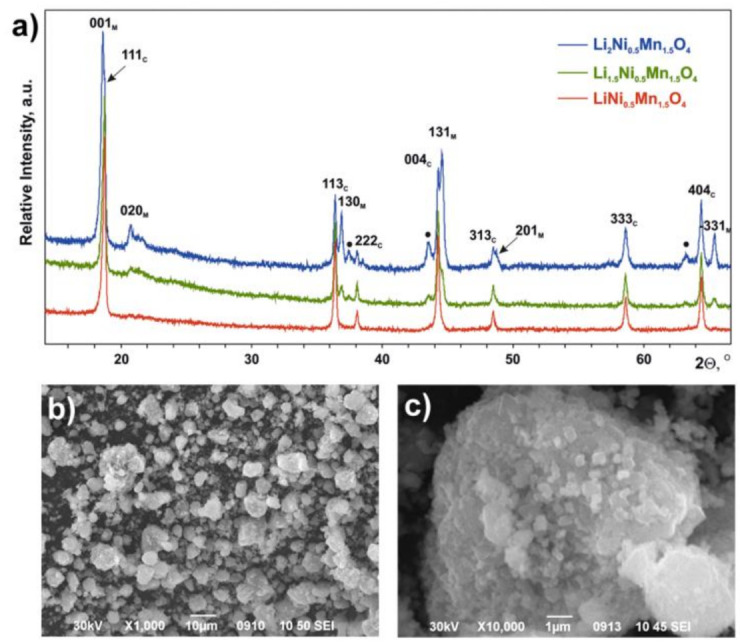
(**a**) Powder X-ray diffraction (PXRD) patterns of the initial samples. The reflections of cubic LiNi_0.5_Mn_1.5_O_4_ (C) and monoclinic Li_1+*x*_M_1−*x*_O_2_ (M) phase are indexed. The reflections of the Li*_z_*M_2−*z*_O_2_ phase are marked by dots. (**b**,**c**) Scanning electron microscopy (SEM) images for the LiNi_0.5_Mn_1.5_O_4_ sample.

PXRD revealed the presence of the cubic spinel phase LiNi_0.5_Mn_1.5_O_4_ (sp.gr. *Fd*$\bar{3}$*m*, *Z* = 8, *a* = 8.1710(3) Å, *V* = 545.54(2) Å^3^) in all synthesized Li_1+*x*_Ni_0.5_Mn_1.5_O_4_ (*x* = 0, 0.5, 1.0) samples. In the case of Li_1.5_Ni_0.5_Mn_1.5_O_4_ and Li_2_Ni_0.5_Mn_1.5_O_4_, the second phase—layered monoclinic Li_1+*x*_M_1−*x*_O_2_ (M = Mn, Ni, sp.gr. *C*2/*m*, *a* = 4.941(1) Å, *b* = 8.542(2) Å, *c* = 5.037(1)Å, *β*= 109.29(2)^o^, *V* = 200.6(1)Å^3^)—was observed with the content increasing with *x* (Figure 1). In addition, an increase in *x* was accompanied by the appearance of and increase in intensity of a few diffraction maxima, which may be assigned to the disordered Li*_z_*M_2−*z*_O_2_ phase (M = Mn, Ni, sp.gr. *R*$\bar{3}$*m*, *Z* = 3, *a* = 2.930(1) Å, *c* = 14.41(3) Å, *V* = 107.1(1)Å^3^) [33,34]. The morphology of all the samples was found to be the same: relatively large (~2–5 µm) spherical aggregates consisting of small (~200–700 nm) crystallites (example for the LiNi_0.5_Mn_1.5_O_4_ material is given in Figure 1).

**Figure 2:**
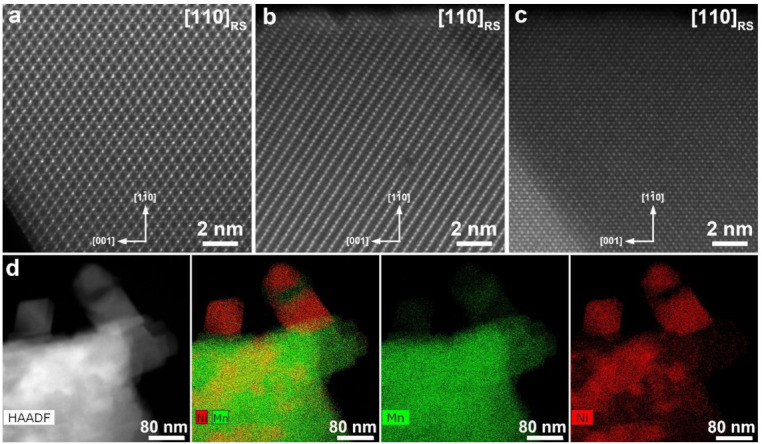
High angle annular darkfield scanning transmission electron microscopy (HAADF-STEM) images of the LiNi_0.5_Mn_1.5_O_4_ (**a**), Li_1+*x*_M_1−*x*_O_2_ (**b**) and Li_z_M_2−z_O_2_ (**c**) phases in the Li_2_Ni_0.5_Mn_1.5_O_4_ sample. The images are taken along the [110] direction of the parent rock-salt (RS) structure. HAADF-STEM image, the color-coded Mn and Ni compositional map and individual energy-dispersive X-ray (EDX) maps of Mn and Ni (**d**) demonstrating three phases with different Ni:Mn atomic ratio.

Atomic-resolution HAADF-STEM images clearly demonstrate co-existence of three different phases in the Li_2_Ni_0.5_Mn_1.5_O_4_ sample (Figure 2). All three are based on the rock-salt structure with the cubic close packing of the oxygen atoms, but differ in the ordering pattern of the Li and d-metal cations. The crystals with the spinel-type ordering (the LiNi_0.5_Mn_1.5_O_4_ phase, Figure 2a), layered ordering (the Li_1+*x*_M_1−*x*_O_2_ phase, Figure 2b) and partially ordered rock-salt phase in which the transition metal cations are preferentially located at the {111} planes (the Li*_z_*M_2−*z*_O_2_ phase, Figure 2c) were observed. EDX compositional maps (Figure 2d) also clearly demonstrates three phases with different Ni:Mn ratio. Quantification of the EDX spectra provides the Ni:Mn ratio of 24(1):76(1), 11(2):89(2) and 82.1(8):17.9(8) for the LiNi_0.5_Mn_1.5_O_4_ phase, Li_1+*x*_M_1−*x*_O_2_ phase and Li*_z_*M_2−*z*_O_2_ phase, respectively.

The phase compositions of the samples were confirmed by Rietveld refinement of PXRD data (Figures S1–S3, the details of the refinement are given in SI) using the values of Ni:Mn ratio for each phase obtained by EDX. The structure of the layered monoclinic phase Li_1+*x*_M_1−*x*_O_2_ can be described as a derivative of the Li_2_MnO_3_structure formed by an alternation of the Li_3_ and Li_1−*x*_M_2+*x*_ layers (M = Mn, Ni) along the *c* axis [5,6,7,8,9,10,11,12,35]. The phase composition used in the refinement corresponds to the formula of Li(Li_0.28_Mn_0.64_Ni_0.08_)O_2_ (it can also be written as Li_1.92_Mn_0.96_Ni_0.12_O_3_, so the composition is close enough to the Li_2_MnO_3_ monoclinic phase). The composition of the third, rhombohedral Li*_z_*M_2−*z*_O_2_ phase can be written as Li_0.5_Ni_1.21_Mn_0.26_O_2_. In the Li_2_Ni_0.5_Mn_1.5_O_4_ sample, the weight fractions of the LiNi_0.5_Mn_1.5_O_4_, Li_1+*x*_M_1−*x*_O_2_ and Li*_z_*M_2−*z*_O_2_ phases were found to be 28(1), 63(1) and 9.0(4) wt.%, respectively.

**Figure 3:**
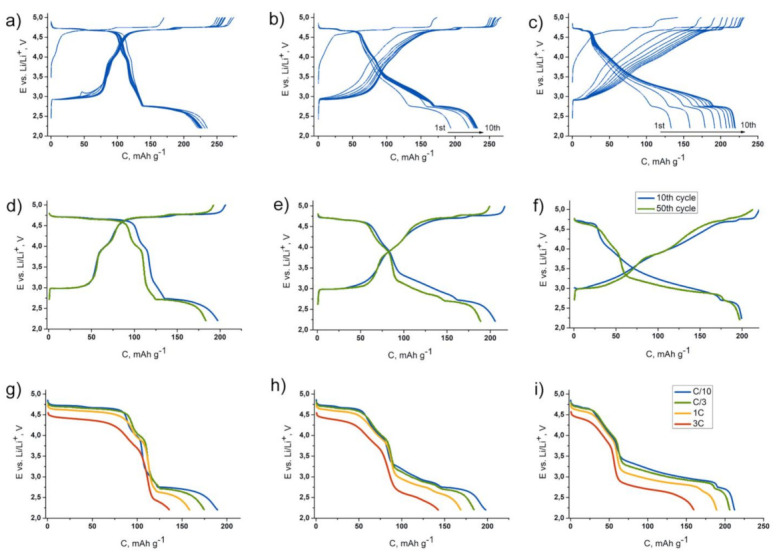
1st–10th charge–discharge curves for the LiNi_0.5_Mn_1.5_O_4_ (**a**), Li_1.5_Ni_0.5_Mn_1.5_O_4_ (**b**) and Li_2_Ni_0.5_Mn_1.5_O_4_ (**c**) samples at C/10 rate; 10th and 50th charge–discharge curves for the same samples at C/3 rate (**d**–**f**); the discharge curves at C/10-3C rate for the same samples after cycling (**g**–**i**).

Electrochemical characterization of the samples (Figure 3) revealed that the cubic spinel LiNi_0.5_Mn_1.5_O_4_ is characterized by two high-voltage plateaus at the first charge, with an average potential of ~4.7 V vs. Li/Li^+^. An additional plateau at ~2.7 V vs. Li/Li^+^ appears at discharge, providing ca. 230 mAh·g^−1^ of the reversible capacity within 2.2–4.9 V potential interval. As it was reported earlier, high-voltage part of the charge–discharge curve is associated with Ni^2+^/Ni^3+^/Ni^4+^ redox transitions, and the low-voltage part with the Mn^3+^/Mn^4+^ transition [22,23,24,25]. Increasing *x* in Li_1+*x*_Ni_0.5_Mn_1.5_O_4_ leads to a decrease in length of the high-voltage and low-voltage plateaus and to appearance of additional capacity within sloping part of the charge–discharge curve situated between 2.8 and 4.7 V vs. Li/Li^+^. Total capacity is approximately the same for all the samples (~230 mAh·g^−1^); however, it grows during several primary cycles in case of Li_1.5_Ni_0.5_Mn_1.5_O_4_ and especially in Li_2_Ni_0.5_Mn_1.5_O_4_. Similar electrochemical behavior was shown earlier for Li_1+*x*_Ni_0.5_Mn_1.5_O_4_ and materials of close compositions [14,15,16,17]. At the moment, there is no unambiguous understanding this phenomenon; authors consider it as higher utilization of the layered phase achieved during cycling. Most likely, it is associated with transformation of the domain structure of the materials following the formation of higher amount of grain boundaries enhancing electrochemical activity. Besides this, the process of oxygen sublattice activation (taking place in the Li-rich cathodes at the fist charge) may be hindered by the presence of spinel phase and therefore several cycles are needed to complete the process. Elongation of the lower part of the sloping plateau region between 3 and 4 V corresponding to Mn^3+^/Mn^4+^ redox in Li-rich oxides, which appears after oxygen evolution, favors such an assumption. Short-term cycling at C/3 charge and discharge rate revealed ca. 10% degradation after 50 cycles for LiNi_0.5_Mn_1.5_O_4_ and Li_1.5_Ni_0.5_Mn_1.5_O_4_ and almost steady capacity for Li_2_Ni_0.5_Mn_1.5_O_4_. The voltage curve of Li_2_Ni_0.5_Mn_1.5_O_4_ undergoes a certain transformation during cycling (Figure 3f). However, it almost does not affect the specific energy density of the material (680 vs. 660 Wh kg^−1^ at 10th and 50th cycles, respectively). The samples demonstrate attractive C-rate capacity retention even after cycling: increasing the current from C/10 to 3C almost does not reduce the capacity associated with the high-voltage part of the charge–discharge curve (Figure 3g–i). However, the lower part (below 3 V vs. Li/Li^+^) definitely demonstrates more sluggish kinetics of Li^+^ incorporation.

**Figure 4:**
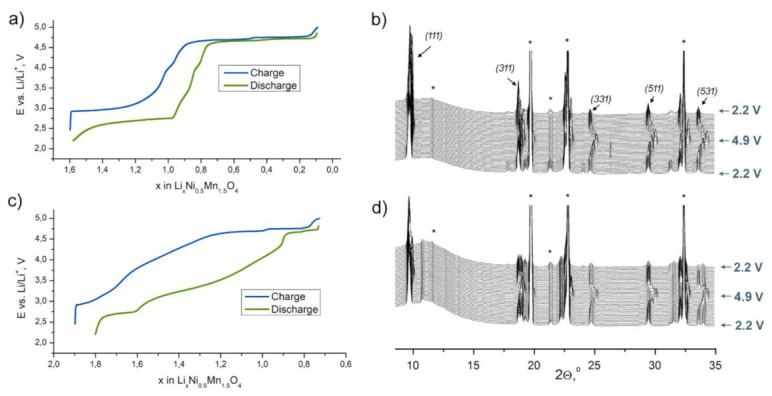
E–x curves and operando synchrotron X-ray powder diffraction (SXPD) patterns of the second charge–discharge cycle for the LiNi_0.5_Mn_1.5_O_4_ (**a**,**b**) and Li_2_Ni_0.5_Mn_1.5_O_4_ (**c**,**d**) materials. The most intense cubic spinel reflections are marked with corresponding *hkl* indices. Asterisks belong to the reflections caused by the cell components.

To analyze structural transitions responsible for the observed electrochemical signatures, we performed SXPD study of LiNi_0.5_Mn_1.5_O_4_ and Li_2_Ni_0.5_Mn_1.5_O_4_ materials in operando regime. Initially, the cells were charged up to 4.9 V and discharged to 2.2 V vs. Li/Li^+^. The obtained diffraction patterns and corresponding E–x curves for the second charge–discharge cycle (2.2–4.9–2.2 V vs. Li/Li^+^) are shown in Figure 4.

**Figure 5:**
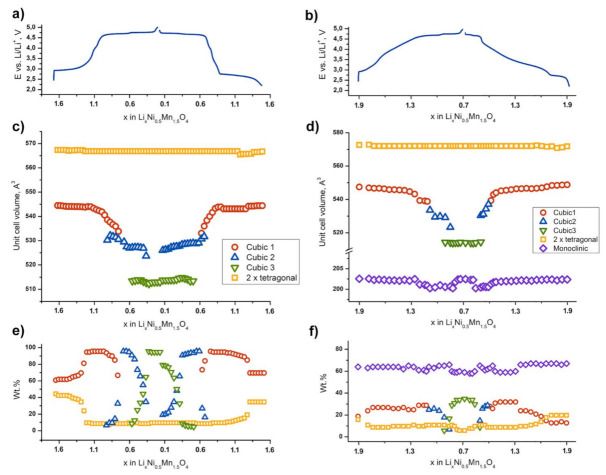
E–x curves and variation of the unit cell volumes and weight fractions of the phases for the LiNi_0.5_Mn_1.5_O_4_ (**a**,**c**,**e**) and Li_2_Ni_0.5_Mn_1.5_O_4_ (**b**,**d**,**f**) samples.

The data on LiNi_0.5_Mn_1.5_O_4_ are consistent with those previously published [23,24,25,26]. During the second charge, three first-order phase transitions with intermediate solid-solution regions (Figure 5) occur: (1) “Tetragonal” ↔ “Cubic 1”at ca. 2.8 V vs. Li/Li^+^; (2) “Cubic 1” ↔ “Cubic 2” at ca. 4.7 V and (3) “Cubic 2” ↔ “Cubic 3” at ca. 4.75 V.

“Tetragonal” phase corresponds to Li_1+*α*_Ni_0.5_Mn_1.5_O_4_ (α ≈ 0.6) (s.g. *I*4_1_/*amd*) formed during the first discharge [36]. The structure of Li_1+*α*_Ni_0.5_Mn_1.5_O_4_ represents a tetragonally distorted spinel, where the deformation is caused by axial elongation of the MnO_6_ octahedra due to the Jahn–Teller effect, induced by partial reduction of Mn^4+^ to Mn^3+^. At the beginning of the second charge (2.7 V), the LiNi_0.5_Mn_1.5_O_4_ cathode material contains about 41 wt.% of Li_1+*α*_Ni_0.5_Mn_1.5_O_4_ (sp.gr. *I*4_1_/*amd*, Z = 4, *a* = 5.7317(6) Å, *c* = 8.637(2) Å, *V* = 283.7(1) Å^3^) and 59 wt.% of the “Cubic 1” phase (sp. gr. *Fd*$\bar{3}$*m*, *Z* = 8, *a* = 8.180(2) Å, *V* = 547.4(1) Å^3^); the latter is similar to the initial spinel. The amount of the tetragonal phase gradually decreases up to ca. 4.0 V vs. Li/Li^+^. The tetragonal phase is characterized by an absence of notable solid solution region. According to our data, some amount of the tetragonal phase (about 5–6 wt.%) remains in the material at the higher potentials and does not participate in redox processes.

The composition of the “Cubic 1” phase can be written as Li_1−*y*_Ni_0.5_Mn_1.5_O_4_, where 0 ≤ *y* < 0.5 [23,24,25,26]. Upon the “Tetragonal” ↔ “Cubic 1” transition, a smooth shrinkage of the cubic spinel cell is observed. Further Li^+^ extraction between 4.0 and 4.7 V and corresponding oxidation of Ni^2+^ to Ni^3+^ forces more drastic “Cubic 1” phase cell volume reduction. The shrinkage of the “Cubic 1” cell is also observed in the “Cubic 1” ↔ “Cubic 2” two-phase region (4.7–4.75 V). In should be noted that the “Cubic 1” phase undergoes the most remarkable cell volume change comparing to other phases in the system: the volume decrease is 5 Å^3^ per one transition metal atom (nearly 2%).

The region of the existence of the “Cubic 2” phase is significantly shorter and relates to the potential range of 4.7–4.8 V. The “Cubic 2” phase preserves the initial cubic spinel structure with the formula of Li_0.5−*y*_Ni_0.5_Mn_1.5_O_4_ [23,24,25,26], where 0.5 ≤ *y*< 0 (sp.gr. *Fd*$\bar{3}$*m*, *Z* = 8, at *y* ≈ 0 *a* = 8.091(6) Å, *V* = 529.5(7) Å^3^). The volume decrease in the “Cubic 2” unit cell upon Li^+^ extraction is also much smaller compared to that of the “Cubic 1” phase and has been found to be 3.5Å^3^ per one transition metal atom (nearly 1.5%). Complete oxidation of Ni^3+^ to Ni^4+^ leads to the “Cubic 3” phase Ni_0.5_Mn_1.5_O_4_. This phase is first revealed as a second phase in the “Cubic 2”–“Cubic 3” two-phase region (in the vicinity of 4.8 V). At 5 V, only the “Cubic 3” phase exists. The lattice constant of the “Cubic 3” phase (sp.gr. *Fd*$\bar{3}$*m*, *Z* = 8, *a* = 7.9955(3) Å, *V* = 511.1(1) Å^3^) does not vary notably; the volume change is as small as 0.2%. It is worth noting that at the end of the “Cubic 1”–“Cubic 2” two-phase region the lattice parameters of both phases are very close to each other while there is no such a behavior for the “Cubic 2”–“Cubic 3” transition.

The described phase transitions demonstrate excellent reproducibility upon the second discharge. As it can be seen in Figure 5a,c, the cell volumes of the phases are similar to the ones upon charge within the value of 3σ.

The behavior of the Li_2_Ni_0.5_Mn_1.5_O_4_-based electrode differs significantly from that of LiNi_0.5_Mn_1.5_O_4_. After the first charge–discharge cycle at the starting point (2.2 V vs. Li/Li^+^), the material consists of cubic spinel “Cubic 1”, tetragonally distorted spinel “Tetragonal” and layered monoclinic Li(Li_0.28_Mn_0.64_Ni_0.08_)O_2_ phase with the phase fraction of more than 60 wt.%. It should be noted that there is no hint of the participation of the rhombohedral Li_0.5_Ni_1.21_Mn_0.26_O_2_ phase in redox processes. Considering the significant overlap of the diffraction maxima of Li_0.5_Ni_1.21_Mn_0.26_O_2_ with the maxima of Al current collector, Li_0.5_Ni_1.21_Mn_0.26_O_2_ was excluded from the refinement of SXPD data and all mass ratio presented hereinafter corresponds to 91% of the active composite fraction.

**Figure 6:**
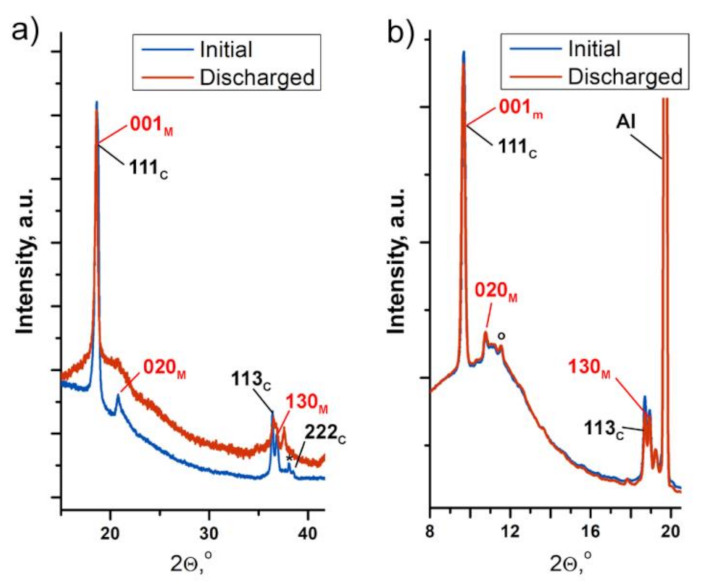
PXRD patterns of the Li_2_Ni_0.5_Mn_1.5_O_4_ electrode at the initial state and after the first charge–discharge cycle collected by means of laboratory (**a**) and synchrotron (**b**) XRD. Reflections of the monoclinic Li(Li_0.28_Mn_0.64_Ni_0.08_)O_2_ phase are marked in red; those for the cubic spinel are in black.

It should be emphasized that no evidence of the degradation/content decrease in the layered monoclinic phase within the Li_2_Ni_0.5_Mn_1.5_O_4_ sample after the first cycle was found using both laboratory (ex situ) and synchrotron (operando) XRD techniques (Figure 6). Recent HRTEM studies of the electrode material with close Li[Ni_1/3_Mn_2/3_]O_2_ composition after cycling also revealed the presence of both monoclinic and spinel phases [17]. In contrast, pure Li_2_MnO_3_ demonstrates different behavior. The irreversible transformation of Li_2_MnO_3_ to the tetragonal spinel-related Li_2_Mn_2_O_4_ phase at the first charge has been reported by Amalraj et al. [37].

Concerning the experiment, at the beginning of the second charge–discharge cycle, the contents of the tetragonal spinel and “Cubic 1” phases are 16 and 19 wt.%, respectively. The phase transitions intrinsic in LiNi_0.5_Mn_1.5_O_4_ are conserved, however, with the dramatic shortening of two-phase transition regions for the cubic phases. There is no two-phase “Cubic 1”—“Cubic 2” region either on charge or on discharge at the second cycle. The two-phase “Cubic 2”—“Cubic 3” transition becomes narrower than that for the LiNi_0.5_Mn_1.5_O_4_ sample. However, the observed cell volume differences for both “Cubic 1” and “Cubic 2” phases are approximately the same, as in the case of LiNi_0.5_Mn_1.5_O_4_. This shortening of two-phase areas may be explained by the higher specific charge–discharge rate due to the lower absolute amount of the cubic phases.

In contrast, the weight fraction of Li(Li_0.28_Mn_0.64_Ni_0.08_)O_2_ as well as its unit cell parameters are nearly stable during the whole charge–discharge cycle except in the high-voltage region (at ca. 4.75 V), where tiny reversible changes can be seen (Figure 5b). In this potential region, the cell volume of Li(Li_0.28_Mn_0.64_Ni_0.08_)O_2_ reduces by 2Å^3^ per one transition metal atom (1%).

Phase transformations taking place during charge–discharge of the Li_1.5_Ni_0.5_Mn_1.5_O_4_ sample are right between the two described materials. However, the monoclinic phase fraction in the Li_1.5_Ni_0.5_Mn_1.5_O_2_ sample is significantly lower than in the Li_2_Ni_0.5_Mn_1.5_O_2_ sample, which hampers the precise determination of its lattice constants taking into account the remarkable reflections overlap. However, the refinement has been done and the results are listed in Tables S1–S3. Variation of unit cell volumes and weight fractions of the involved phases are shown in Figure S4.

## 4. Discussion

As it can be seen from Figure 3a,c, reversible capacities of the LiNi_0.5_Mn_1.5_O_4_ spinel and spinel-layered Li_2_Ni_0.5_Mn_1.5_O_4_ materials at the second charge–discharge cycle are ~220 and ~170 mAh·g^−1^, respectively. Since the mass fraction of the spinel-type phases in the Li_2_Ni_0.5_Mn_1.5_O_4_-based electrode is almost steady during charge–discharge process and corresponds to 35–40%, we can assume that appr. 50% of the capacity obtained during the second cycle belongs to the monoclinic layered oxide Li(Li_0.28_Mn_0.64_Ni_0.08_)O_2_. The oxidation state of Mn in this oxide is +4, as in Li_2_MnO_3_, so we may conclude that anionic redox is predominantly responsible for the electrochemical activity of this component.

Better cycling stability of the Li_2_Ni_0.5_Mn_1.5_O_4_ material compared to LiNi_0.5_Mn_1.5_O_4_ is most probably related to improved mechanical stability: the lower volume fraction of the spinel-type phases with high specific volume change on the charge/discharge leads to a decrease in total volume variation. This finding is in a good agreement with the earlier studies on LiNi_0.5_Mn_1.5_O_4_ and LiNi_0.5_Mn_1.5_O_4_-Li_2_MnO_3_ composites by means of HRTEM [38]. Lu et al. observed significant pulverization of LiNi_0.5_Mn_1.5_O_4_ after cycling within the extended voltage range of 2.0–5.0 V vs. Li/Li^+^. Microstructure of the electrode consisting of LiNi_0.5_Mn_1.5_O_4_ and monoclinic domains was found to be much more stable, as well as its electrochemical characteristics upon cycling [38].

**Figure 7:**
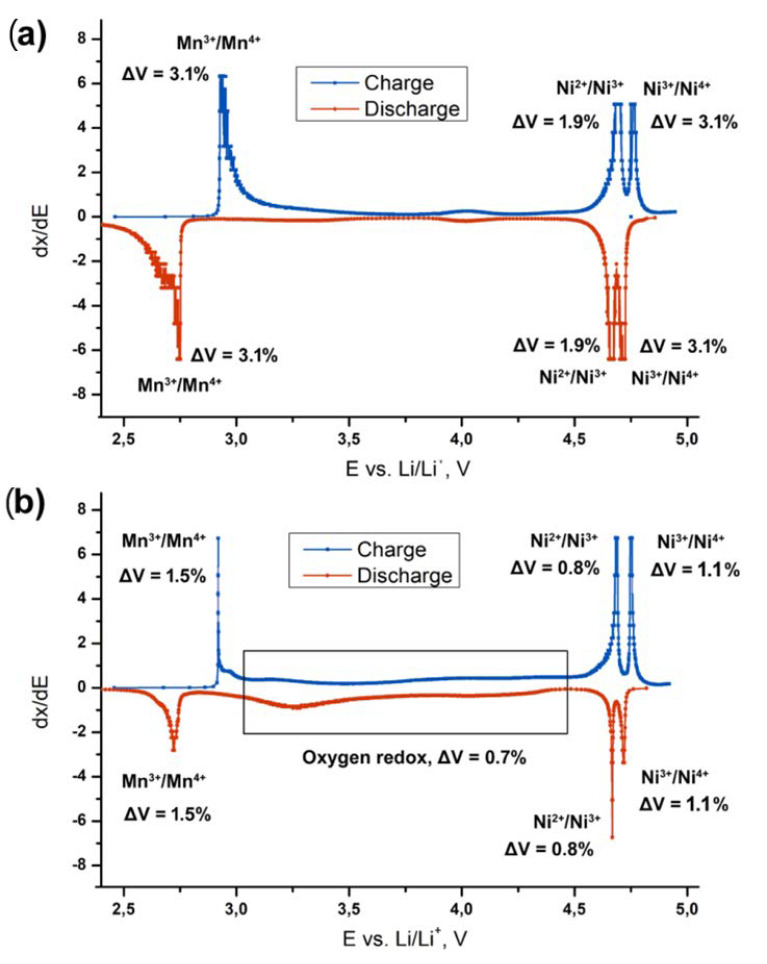
Differential capacity curves for the (**a**) LiNi_0.5_Mn_1.5_O_4_ and (**b**) Li_2_Ni_0.5_Mn_1.5_O_4_ electrodes, the corresponding redox transitions [23,24,25,26] and volume changes.

Based on the data presented in Figure 5 and literature data on the redox activity of Ni and Mn cations, we suggest a tentative scheme of the electrochemical transitions occurring at (De)lithiation of LiNi_0.5_Mn_1.5_O_4_ and Li_2_Ni_0.5_Mn_1.5_O_4_, and corresponding volume changes of the overall electrode composite (determined taking into account the volumetric changes during phase transitions and the mass fractions of the involved phases). The scheme is illustrated in Figure 7. The overall estimated volume change of the LiNi_0.5_Mn_1.5_O_4_ electrode is twice larger than that for Li_2_Ni_0.5_Mn_1.5_O_4_, as can be seen from the Figure 6, and mainly caused by Mn^3+^/Mn^4+^ and Ni^3+^/Ni^4+^ transitions at 2.8 and 4.75 V, respectively. Since the mass fraction of the spinel-type phases in Li_2_Ni_0.5_Mn_1.5_O_4_ does not exceed 30%, these transitions do not strongly affect overall volume change. Therefore, this composite material appears to be mechanically sustainable, which gives another reason to consider it promising for next-generation LIBs.

## 5. Conclusions

Cobalt-free “spinel-layered” composites of the Li_1+*x*_Ni_0.5_Mn_1.5_O_2_” (*x* = 0, 0.5, 1) composition demonstrate attractive electrochemical performance with capacity exceeding 200 mAh·g^−1^ within 2.2–4.9 V vs. Li/Li^+^. Operando synchrotron X-ray diffraction studies revealed complex phase transition mechanisms including tetragonal, three cubic and monoclinic phases. Although phase transitions for the spinel-type phases in the Li_2_Ni_0.5_Mn_1.5_O_4_ cathode material remain almost the same as in the case of LiNi_0.5_Mn_1.5_O_4_, they are associated with only half of the observed capacity, which means that the layered Li-rich Li(Li_0.28_Mn_0.64_Ni_0.08_)O_2_ phase significantly contributes to the electrochemical activity of the whole composite. The redox process associated with Li(Li_0.28_Mn_0.64_Ni_0.08_)O_2_ is characterized by very small unit cell volume change of 0.7% and therefore provides better mechanical stability of the “spinel-layered” composites during electrochemical operation.

## Data Availability

Not applicable.

## Supplementary Materials

The following are available online at https://www.mdpi.com/article/10.3390/nano11061368/s1, Figures S1–S3; Tables S1–S3.
