# Supplementary material for: Phase Transitions in the “Spinel-Layered” Li1+xNi0.5Mn1.5O4 (x = 0, 0.5, 1) Cathodes upon (De)lithiation Studied with Operando Synchrotron X-ray Powder Diffraction
Source: Nanomaterials (Basel). 2021 May 21;11(6):1368. doi: 10.3390/nano11061368 (PMC8224351; doi:10.3390/nano11061368)
Supplement: Supplementary file 1 [file nanomaterials-11-01368-s001.zip › Supplementary/Revised_SM.pdf]

Supplementary Materials for

Phase transitions in the "spinel-layered" $Li_{1+x}Ni_{0.5}Mn_{1.5}O_4$ (x = 0, 0.5, 1) cathodes upon (de)lithiation studied with *operando* synchrotron X-ray powder diffraction.

Oleg A. Drozhzhin[1,2*], Anastasia M. Alekseeva[1], Vitaly A. Shevchenko[1,2], Dmitry Chernyshov[3,4], Artem M. Abakumov[2], Evgeny V. Antipov[1,2]

[1] Department of Chemistry, Lomonosov Moscow State University, 119991 Moscow, Russian Federation

[2] Skoltech Center for Energy Science and Technology, Skolkovo Institute of Science and Technology, Nobel str. 3, 143026 Moscow, Russian Federation

[3] Swiss–Norwegian Beamlines, European Synchrotron, 71 Rue des Martyrs, Grenoble, 38043, France

[4] Peter the Great St. Petersburg Polytechnic University, 29 Polytekhnicheskaya St, Saint-Petersburg, 195251, Russia

[*] Correspondence: drozhzhin@elch.chem.msu.ru

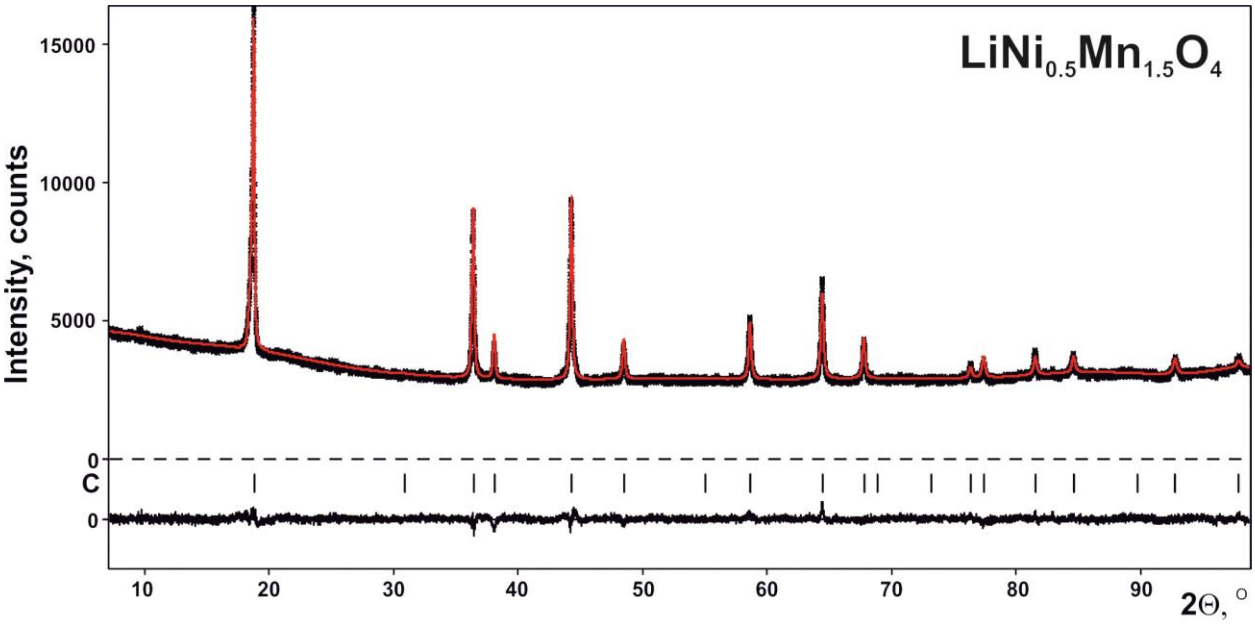

Figure S1. Experimental, calculated (red) and difference PXRD (λ = 1.540598 Å) patterns for $LiNi_{0.5}Mn_{0.5}O_4$ sample. PXRD data were refined by Rietveld method using JANA 2006 software as $LiNi_{0.5}Mn_{0.5}O_4$ cubic spinel (C) (sp. gr. $Fd\bar{3}m$, Z = 8, a = 8.1710(3) Å, V = 545.54(2) Å^3, R_F = 0.047, R_P = 0.018, wR_P = 0.023, GOF = 1.31). The used structure model was the follow: Li 8*a*

(⅛,⅛,⅛) 1[1]; Ni/Mn 16*d* (½, ½, ½) 0.25/0.75[1]; O 32*e* (0.2632, 0.2632, 0.2632) 1[1] [S1]. The 16*d* site occupancy was fixed in accordance with Ni:Mn ratio founded by EDX.

[1]site occupancy

[S1] W. Branford, M. A. Green, D. A. Neumann, Chem. Mat., 14 (2002) 1649–1656.

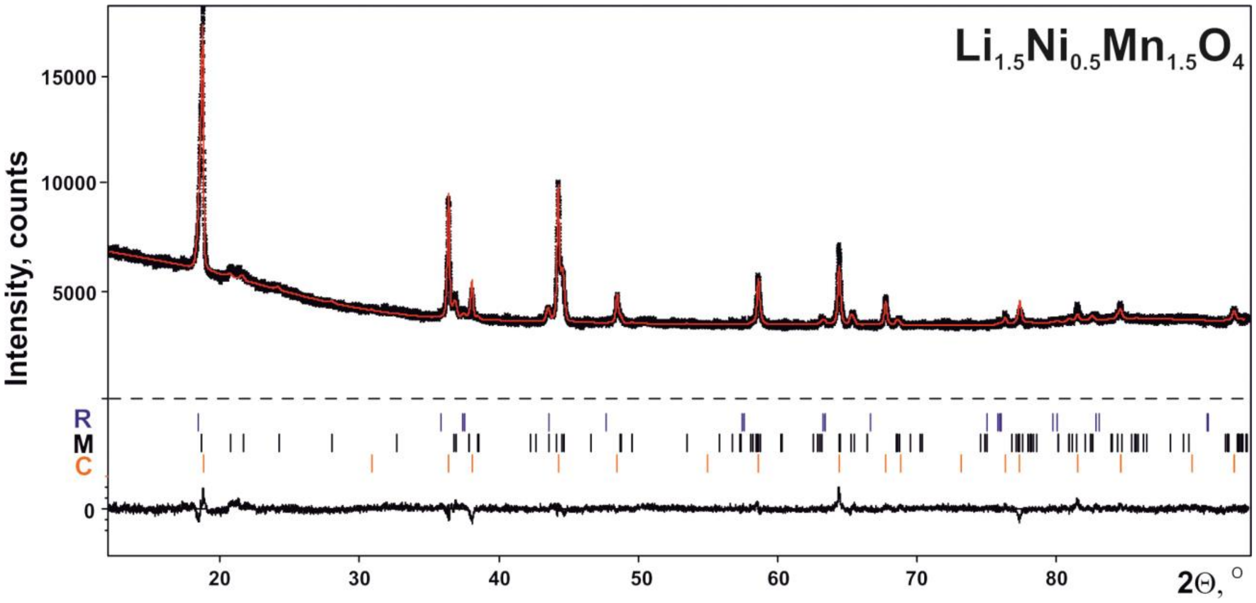

Figure S2. Experimental, calculated (red) and difference PXRD (λ = 1.540598 Å) patterns for $Li_{1.5}Ni_{0.5}Mn_{0.5}O_4$ sample. PXRD data were refined by Rietveld method using JANA 2006 software as three phase mixture of:

C - $LiNi_{0.5}Mn_{0.5}O_4$ (sp. gr. $Fd\bar{3}m$, Z = 8, a = 8.1720(3) Å, V = 545.7(1) Å^3, R_F = 0.088, 60(2) wt. %),

M – $Li(Li_{0.28}Mn_{0.64}Ni_{0.08})O_2$ (sp. gr. $C2/m$, Z = 4, a = 4.950(3) Å, b = 8.543(5) Å, c = 5.028(2) Å, β = 109.25(5)°, V = 200.7(1) Å^3, R_F = 0.098, 34(2) wt. %),

R – $Li_{0.5}Ni_{1.21}Mn_{0.26}O_2$ (sp. gr. $R\bar{3}m$, Z = 3, a = 2.932(3) Å, c = 14.41(3) Å, V = 107.3(1) Å^3, R_F = 0.11, 5.2(6) wt. %) with R_P = 0.018, wR_P = 0.024, GOF = 1.58.

The used structure models were the follow:

$LiNi_{0.5}Mn_{0.5}O_4$: similar to aforementioned one.

$Li(Li_{0.28}Mn_{0.64}Ni_{0.08})O_2$: Li1/Mn1/Ni1 2*b* (0, ½, 0) 0.64/0.32/0.04[1]; Li2/Mn2/Ni2 4*g* (0, 0.1687, 0) 0.1/0.81/0.1[1]; Li3 2*c* (0, 0, ½) 1[1]; Li4 4*h* (0, 0.319, ½) 1[1]; O1 4*i* (0.224, 0, 0.225) 1[1]; O2 8*j* (0.253, 0.323, 0.2275) 1[1] [S2].

At the first refinement attempt the ordered Li_2MnO_3 structure model (Li1 2*b*; Li2 2*c*; Li3 4*h*, Mn1 4*g*; O1 4*i*; O2 8*j*) was used. Strong disagreement for experimental and calculated intensities of several diffraction maxima together with EDX data was the reason to use the disordered model with Li/M (M = $Mn_{0.89}Ni_{0.11}$) statistic occupation of cation sites [S2]. The refined values of atomic displacement parameters were used as a hint for presence of Li/M statistic occupation for certain positions. Finally, Li/Mn/Ni joint occupancy for 2*b* and 4*g* sites was refined.

$Li_{0.5}Ni_{1.21}Mn_{0.26}O_2$: Li1/Ni1/Mn1 3*b* (0, 0, ½) 0.252/0.600/0.132[1]; Li2/Ni2/Mn2 3*a* (0, 0, 0) 0.252/0.600/0.135[2], O 6*c* (0, 0, 0.2458) 1[1] [S3].

The site occupancies were fixed in accordance with EDX data.

[1]site occupancies

[S2] A. Boulineau, L. Croguennec, C. Delmas, F. Weill, Chem. Mat., 21 (2009) 4216–4222.

W. Li, J.N. Reimers, J.R. Dahn, Phys. Rev. B, 46 (1992) 3236–3246.

[S3] W. Li, J.N. Reimers, J.R. Dahn, Crystal structure of $Li_xNi_{2-x}O_2$ and a lattice-gas model for the ordered-disordered transition, Phys. Rev. B, 46 (1992) 3236–3246.

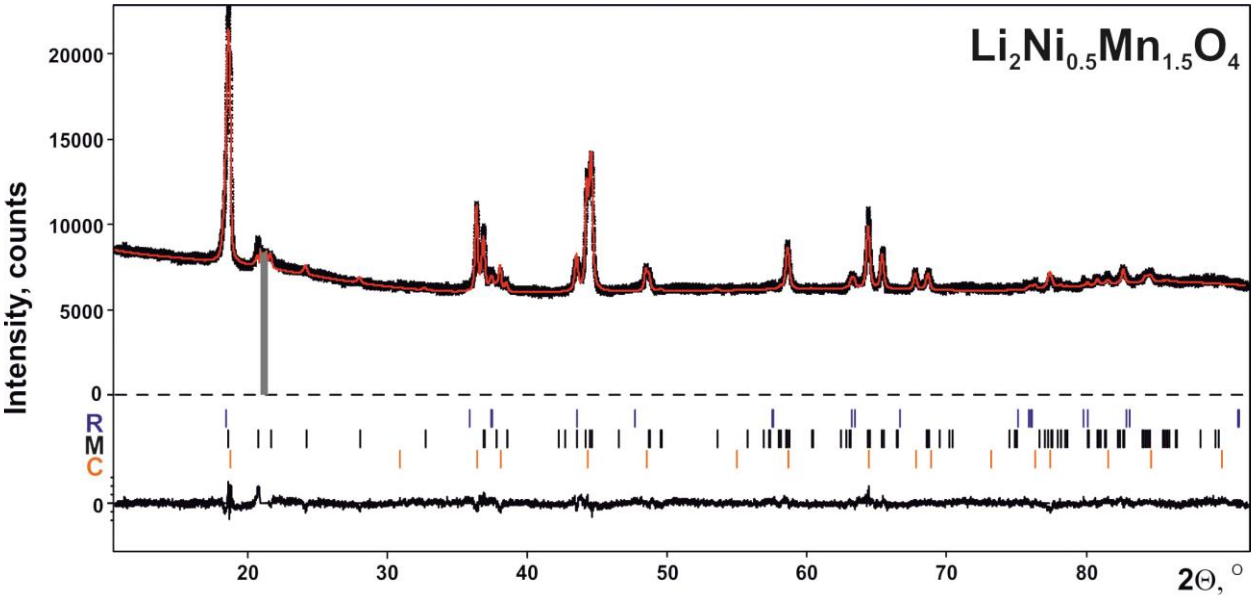

Figure S3. Experimental, calculated (red) and difference PXRD (λ = 1.54598 Å) patterns for $Li_2Ni_{0.5}Mn_{0.5}O_4$ sample. PXRD data were refined by Rietveld method using JANA 2006 software as three phase mixture of:

C – $LiNi_{0.5}Mn_{0.5}O_4$ cubic spinel (sp. gr. $Fd\bar{3}m$, Z = 8, a = 8.1692(4) Å, V = 545.2(1) Å^3, R_F = 0.071, 28(1) wt. %),

M – $Li(Li_{0.28}Mn_{0.64}Ni_{0.08})O_2$ (sp. gr. $C2/m$, Z = 4, a = 4.940(1) Å, b = 8.542(2) Å, c = 5.034(1) Å, β = 109.28(2)°, V = 200.5(1) Å^3, R_F = 0.080, 63(1) wt. %.

R – $Li_{0.5}Ni_{1.21}Mn_{0.26}O_2$ (sp. gr. $R\bar{3}m$, Z = 3, a = 2.930(1) Å, c = 14.41(1) Å, V = 107.1(1) Å^3, R_F = 0.073, 9.0(4) wt. %) with R_P = 0.017, wR_P = 0.020, GOF = 1.68.

The used structure models were similar to aforementioned ones.

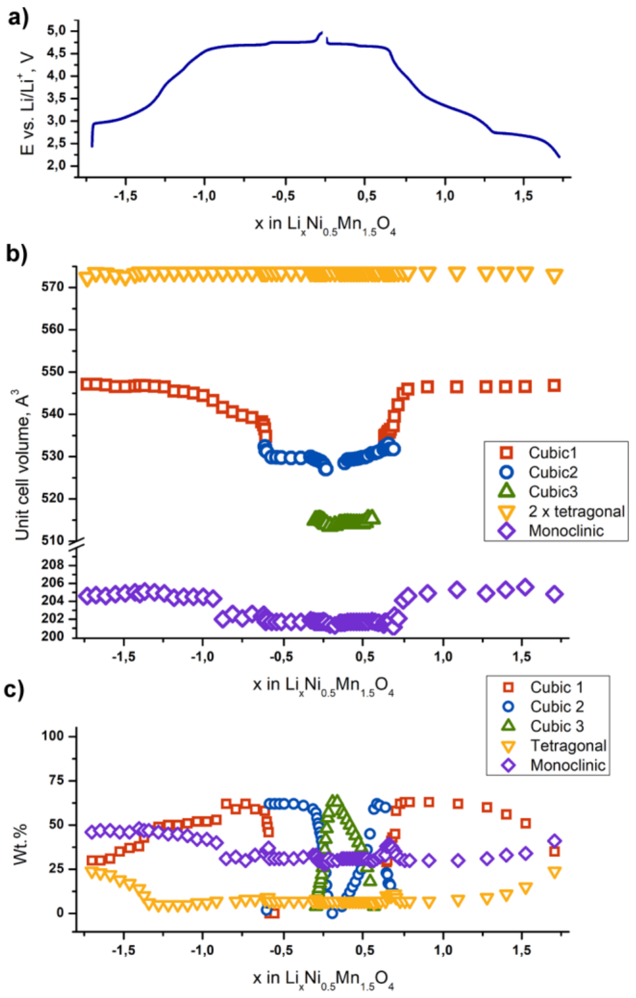

Figure S4. E–x curves (a) and variation of the unit cell volumes (b) and weight fractions of the phases (c) for the $Li_{1.5}Ni_{0.5}Mn_{1.5}O_4$ sample
